# A Multiclass Classification Model for Tooth Removal Procedures

**DOI:** 10.1177/00220345221117745

**Published:** 2022-09-09

**Authors:** W.M. de Graaf, T.C.T. van Riet, J. de Lange, J. Kober

**Affiliations:** 1 Mechanical, Maritime and Materials Engineering (3ME), Department of Cognitive Robotics, Delft University of Technology, Delft, The Netherlands; 2 Amsterdam University Medical Center (AUMC), Department of Oral and Maxillofacial Surgery, University of Amsterdam, Amsterdam, The Netherlands; 3 Academic Center for Dentistry Amsterdam (ACTA), University of Amsterdam, Amsterdam, The Netherlands

**Keywords:** machine learning, education, operative, models, tooth extraction, evidence-based dentistry

## Abstract

Surprisingly little is known about tooth removal procedures. This might be due to the difficulty of gaining reliable data on these procedures. To improve our understanding of these procedures, machine learning techniques were used to design a multiclass classification model of tooth removal based on force, torque, and movement data recorded during tooth removal. A measurement setup consisting of, among others, robot technology was used to gather high-quality data on forces, torques, and movement in clinically relevant dimensions. Fresh-frozen cadavers were used to match the clinical situation as closely as possible. Clinically interpretable variables or “features” were engineered and feature selection took place to process the data. A Gaussian naive Bayes model was trained to classify tooth removal procedures. Data of 110 successful tooth removal experiments were available to train the model. Out of 75 clinically designed features, 33 were selected for the classification model. The overall accuracy of the classification model in 4 random subsamples of data was 86% in the training set and 54% in the test set. In 95% and 88%, respectively, the model correctly classified the (upper or lower) jaw and either the right class or a class of neighboring teeth. This article discusses the design and performance of a multiclass classification model for tooth removal. Despite the relatively small data set, the quality of the data was sufficient to develop a first model with reasonable performance. The results of the feature engineering, selection process, and the classification model itself can be considered a strong first step toward a better understanding of these complex procedures. It has the potential to aid in the development of evidence-based educational material and clinical guidelines in the near future.

## Introduction

Aulus Cornelius Celsus (c. 25 BC–AD 50) described tooth removal procedures for the first time in his “De Medicina” with an instruction: “it is to be shook; which must be continued till it move easily” (Celsus 1814). In modern textbooks, descriptions of these complex procedures have not changed significantly (Stegenga 2013). Being one of the oldest and most commonly performed surgical procedures worldwide, the lack of scientific progress in this field is surprising. Scientific attempts to increase our understanding of these procedures are relatively rare, heterogeneous, and mostly focused on extraction forces (Ahel et al. 2006; Cicciù et al. 2013; Dietrich et al. 2020; Sugahara et al. 2021). Analyzing different aspects of tooth removal, especially in clinical situations, requires measurements of subtle movements and high forces in a confined space (intraorally), which might explain the knowledge gap in this field (van Riet et al. 2020).

Through a collaboration between computer scientists, mechanical engineers, and oral- and maxillofacial (OMF) surgeons, a setup was designed to measure different aspects of tooth removal procedures (van Riet et al. 2020). With the use of compliant robotics, data were gathered on (rotational) forces and movements in all their dimensions and directions, in high detail, and at a high frequency. While individual parts of data can be explained and understood with traditional statistical methods, analyzing their combination is complex. Machine learning can be particularly useful to understand and analyze complex or large data sets with many variables, in which it has the potential to detect relationships. It can be considered essential to make use of the data as a whole. A classification model is an example of machine learning technology that consists of an algorithm capable of predicting which tooth was removed based on a variety of complex data. It could aid in finding which variables are most relevant in tooth removal procedures and to evaluate how procedures differ between certain teeth. This can be of use for, among others, the development ofevidence-based education material.

The goal of this project was to build and validate a first and exploratory classification model for tooth removal based on force, torque, and movement data. By evaluating which variable (or “feature”) is selected by the algorithm, a unique insight in this ancient procedure is presented. This article describes our methods of data collection using robot technology, the feature design process, and the model’s performance.

## Materials and Methods

### Data Collection

**Figure 1.**
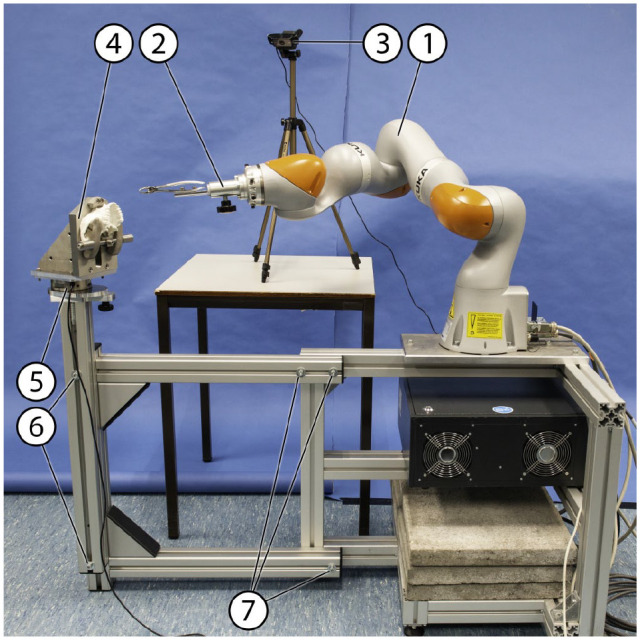
Overview of the setup with a 3-dimensional printed upper jaw in situ. (1) Passive robot arm, (2) forceps holding device, (3) video camera, (4) upper jaw holding device (the lower jaw holding device not shown in this figure), (5) 6-axis force/torque sensor, (6) bolts to change vertical position, and (7) bolts to change horizontal position.

An ex vivo measurement campaign was designed to collect relevant data. Seven fresh-frozen cadavers were obtained from the clinical anatomy and embryology section of the Department of Medical Biology of the Amsterdam University Medical Center (Amsterdam UMC). The donation process was in accordance with Dutch legislation and the regulations of the medical ethical committee of the Amsterdam UMC. Extractions were performed by 3 senior oral and maxillofacial surgeons. An extensive measurement setup was used, as described in more detail in previous work (van Riet et al. 2020). An overview of the setup is presented in **Figure 1**. In short, data on position, orientation, and movements were gained through a compliant robot arm (LBR iiwa 7 R800; KUKA) passively following the movements of an OMF surgeon (frequency 100 Hz). A 6-axis force/torque (FT) sensor (ATI Industrial Automation 16-bit Delta transducer) was used to register forces and torques at 20 Hz. An open-source framework was used for integration of the components (Robot Operating System [ROS]). A custom graphical user interface (GUI) was designed to allow for the addition of metadata on the experiments itself (e.g., reason in case of any failed measurements) and on the clinical status of the teeth (e.g., restorative and periodontal state). In total, the setup gathers 13-dimensional time series for each individual tooth removal procedure. Six-dimensional time series from the force/torque sensor consist of 3 dimensions (“XYZ”) for both forces and torques. A further 7-dimensional time series is gathered from the robot arm—3 dimensions for the position of the end-effector (“XYZ”) and a 4-dimensional representation of the orientation of the end-effector in quaternions (Challis 2020). For data analysis, Python was used (Python Language Reference, version 3.9; Python Software Foundation) (van Rossum and Drake 1995) and the Scikit-learn 1.0.1 module (Pedregosa et al. 2011). A calibration step was performed just prior to each experiment to determine the position and orientation of each tooth (van Riet et al. 2020). Reporting guidelines were used to structure this report (Luo et al. 2016; Schwendicke et al. 2021).

### Preprocessing the Data

Because each measurement started and stopped manually, some meaningless data were gathered just prior and after each experiment. Raw data were therefore manually trimmed, using the custom user interface, directly after each experiment. Using data from the calibration step, raw data from the force/torque sensor and robot arm were mathematically transformed from their own reference frames to the clinically relevant tooth frame (van Riet et al. 2020). This results in 1 unified reference frame in which, for example, a positive value on the X-axis in force and movement data are both in a buccal direction. A negative value on the X-axis means a force or movement in the lingual direction. This also holds for the Y-axis (mesial/distal or proximal/distal along the dental arch curve) and Z-axis (intrusion/extrusion). Time-series data were filtered for noise reduction purposes with a low-pass Butterworth filter (Challis and Kitney 1983). Data of the force/torque sensor (20 Hz) were upsampled to match the frequency of the movement data (100 Hz) using a standard fast Fourier transformation (Yoganathan et al. 1976).

### Feature Design and Selection

Based on the existing force/torque and movement data, additional variables—so-called features—can be computed. These features can be best compared to the (independent) “variables” we know from traditional statistics. They were designed in multiple brainstorming sessions between computer scientists and OMF surgeons. An effort was made to design clinically interpretable features (e.g., rotational velocity or peak forces/torques in every direction). For a complete overview of all features, see Appendix Table 1. Each of these features has its own predictive power to distinguish between different classes of teeth.

Teeth were grouped together as “classes” to optimize model performance for a small data set. To ease the clinical interpretability of this model, 4 classes were chosen as an output for the model. These classes were the same for both the upper (U) and the lower (L) jaw: incisors (U1/U2, L1/L2), cuspids (U3, L3), bicuspids (U4/U5, L4/L5), and molars (U6/U7,L6/L7).

The goal of feature selection is to determine what features should be included in order to optimally classify tooth removal procedures with a minimum set of features (Bursac et al. 2008; Brick et al. 2017). Several approaches are available to select the most important features, of which “regularization” is one (Bishop 2006). A model including a regularization term trades off simplicity and performance by weighting different features. The model is simplified by discarding uninformative features at the cost of a reduction in classification accuracy. This way, only features with high importance will remain. For this study, logistic regression with L2 (or “ridge regression”) regularization was used. L2 regularization was chosen over L1 (or “lasso regression”) because it is more suitable to avoid overfitting of a model. In contrast to L1 regularization, features are not removed from the model in L2, but it tends to reduce extreme weights, leading to a more even distribution of the weight of the features. The actual selection is then performed by applying a threshold for feature importance, which, in our study, was chosen to be the mean of the overall feature importance (Pedregosa et al. 2011).

### Designing a Classification Model

Because features can differ in terms of scale, standardization (i.e., variance scaling) of the features was performed to even out their scales. In the standardization process, every feature is scaled down to a mean of zero and a standard deviation of 1. It prevents the algorithm mistakenly giving importance to features that have larger scales.

As a classification algorithm, Gaussian naive Bayes (GNB) was used. It is a probabilistic machine learning algorithm that can be used for a variety of classification tasks. Our data set has limited size and high variance, with an approximately Gaussian (or normal) distribution. Naive Bayes classifiers are well known for their performance on problems with a small amount of training data (Zhang 2004), while logistic regression models—used for feature selection in this article—are more prone to overfitting for such problems. Accuracy, precision, recall, and F1 score were calculated for each tooth class to evaluate the model performance. To reduce the risk of selection bias and to more accurately estimate the model’s predictive performance, a stratified 4-fold cross-validation was performed. In this cross-validation, 4 random subsamples of data are used to calculate the performance metrics with the same class proportions (stratified) due to the small sample size.

### Data Availability

Data required to reproduce these findings are available to download from https://www.doi.org (digital object identifier: 10.4121/19665990).

## Results

### Clinical Characteristics

A total of 127 experiments were performed on 7 fresh-frozen Caucasian specimens. In 110 (86.6%), experiment data were successfully recorded. A heterogeneous group of teeth in terms of restorative and periodontal states was included (Appendix Table 2).

### Feature Design

**Figure 2.**
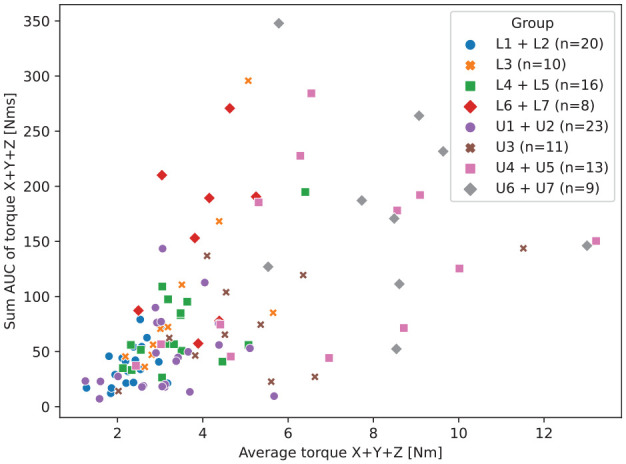
Plot of all 110 datapoints showing the relationship between 2 features, the AUC of the torque magnitude (sum of torques on all 3 axes combined) and average torques (on all 3 axes combined). AUC, area under the curve; L, lower; Nm, Newton meter; Nms, Newton meter second; U, upper.

In total, 75 features were designed, of which 33 remained after regularization. An overview of these selected features is given in Table 1. The relationship between 2 strong prediction features, the sum of delivered torques and average torques on all 3 axes, is shown in Figure 2. It is an example of how these features can be used to distinguish different classes of teeth. While the sum of torques in all directions can be high for both upper and lower jaw bicuspids and molars, it seems that average torques in the lower jaw are higher in the dorsal area compared to the upper jaw. Also, in both upper and lower jaw incisors, average torques did not reach above 6 Nm.

**Table 1.** An Overview of Selected Features.

Force and Torque Data Features	Axis	Direction	Number (*n* = 17)
Sum (AUC) of forces (Ns)	X + Y + Z	All	4
	X-axis (+)	Buccal	
	Y-axis (–)	Distal	
	Z-axis (–)	Extrusion	
Average forces (N)	X + Y + Z	All	2
	Y-axis (+)	Mesial	
Sum (AUC) of torques (Nms)	X + Y + Z	All	4
	Y-axis (+)	Buccoversion	
	Z-axis (+)	Mesiobuccal rotation	
	Z-axis (–)	Mesiopalatal-lingual rotation	
Average torques (Nm)	X + Y + Z	All	4
	X-axis (+)	Mesial angulation	
	Y-axis (+)	Buccoversion	
	Z-axis (+)	Mesiopalatal-lingual rotation	
Peak forces (N)	X + Y + Z	All	1
Peak torque (Nm)	X + Y + Z	All	1
Percentage of amount of force, relative to the sum of all 3 axes (%)	Z-axis	Intrusion/extrusion	1
Rotational Data Features	Axis	Direction	Number (*n* = 16)
Percentage of amount of rotation, relative to the sum of all 3 axes	Y-axis	Bucco-palato/linguoversion	2
	Z-axis	Mesiopalatal-lingual rotation	
Maximum rotations (deg)	Y-axis (+)	Buccoversion	2
	Z-axis (–)	Mesiopalatal-lingual rotation	
Average rotations (deg)	Y-axis (+)	Buccoversion	4
	Y-axis (–)	Linguoversion	
	Z-axis (+)	Mesiobuccal rotation	
	Z-axis (–)	Mesiopalatal-lingual rotation	
Variation of rotation on a single axis (deg)	Z-axis	Mesiobuccal/mesiopalatal-lingual rotation	1
Maximum rotational velocity (deg/s)	Y-axis (+)	Buccoversion	4
	Y-axis (–)	Linguoversion	
	Z-axis (+)	Mesiobuccal rotation	
	Z-axis (–)	Mesiopalatal/lingual rotation	
Variation of rotational velocity on a single axis (deg/s)	X-axis	Mesial-distal angulation	3
	Y-axis	Bucco-palato/linguoversion	
	Z-axis	Mesiobuccal-mesiopalatal/lingual rotation	

X + Y + Z represents the sum of all axes. In case of rotational data (torques and all rotational data features), a rotation around the mentioned axis takes place.

(+), only positive values on specified axis; (–), only negative values on specified axis; AUC, area under the curve; deg, degree; deg/s, degrees per second; N, Newton; Nms, Newton meter second; Ns, Newton second; X-axis, buccolingual; Y-axis, mesiodistal; Z-axis, longitudinal axis.

### Model Performance

**Figure 3.**
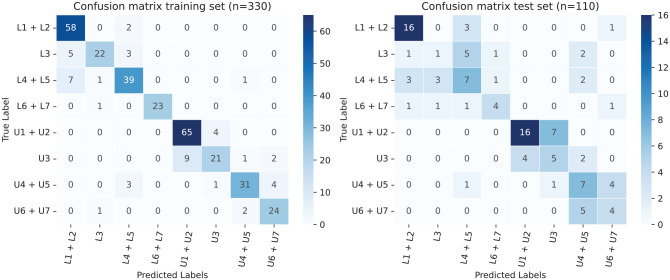
Confusion matrix in which the cumulative predictions of the 4-fold cross-validation are presented. The training set, containing 330 teeth, is shown on the left side and the test set containing 110 on the right side. The center diagonal represents correctly predicted labels. L, lower; U, upper.

A summary of the performance of the model is given in Table 2. On average, the accuracy was 86% in the training set and 54% in the test set (unseen data). The data are presented in 2 confusion matrices, which show the cumulative results of the 4 subsamples (Fig. 3). In the test set (unseen data), in 104 out of 110 experiments (95%), the correct jaw (upper/lower) was classified. Also, 97 experiments (88%) were either correctly classified or as a neighboring class.

**Table 2.** Performance Metrics of the Classification Model for Both Training and Test Sets.

Characteristic	Subsample 1, %	Subsample 2, %	Subsample 3, %	Subsample 4, %	Average, %
Training set	*n* = 82	*n* = 82	*n* = 83	*n* = 83	
Accuracy	84	88	86	86	86
Precision	87	90	88	87	88
Recall	84	88	86	86	86
F1 score	85	88	86	86	86
Test set	*n* = 28	*n* = 28	*n* = 27	*n* = 27	
Accuracy	64	54	56	44	54
Precision	84	61	65	44	55
Recall	64	54	56	44	54
F1 score	71	53	57	47	57

## Discussion

The goal of this project was to build a classification model for tooth removal. The measurement campaign was described in short as well as the process of feature design. A classification model, which is capable of predicting tooth classes based on force and movement data, was presented.

The overall accuracy of the model, after cross-validation in 4 subsamples of data, was 86% in the training set and 54% in the test set (unseen data). The model correctly predicts the (upper or lower) jaw in 95% of the experiments. In 88%, it predicts either the correct class or a class of neighboring teeth. This means that, based on variables derived from complex force and movement data, the algorithm is capable of determining to which “tooth class” a measurement belongs to, with reasonable performance. These results seem reasonable, given the heterogeneity in the data due to surgeon and patient factors in combination with a relatively small data set to train the model on. Another factor that might explain the relative low accuracy and precision might be an incorrect class selection. If tooth removal strategies are similar for certain classes, for example, bicuspids and cuspids in the lower jaw, the model’s performance will decrease. It could be valuable, in future research and for educational purposes, to let the model optimize the class selection instead (i.e., perform clustering). An important finding in this study is that the collected data are of sufficient quality to use for modern learning techniques. Further data collection is necessary to allow for the use of clinical metadata and to further increase the models’ performance and generalizability.

The feature design and selection processes are an essential part of building a classification model. The evaluation of which features are most relevant for the algorithm to classify an experiment is an important first step to improve our fundamental understanding of these complex procedures. While a detailed discussion on the relevance of each feature falls outside the scope of this article, a few key findings are highlighted here. In terms of force and torque data, in each group of features, the sum of forces and torques on all 3 axes combined was selected. This means that the sum of all forces and torques in an experiment is descriptive for classification purposes, rather than forces in individual directions. When looking at rotational and velocity data, features containing rotation around the Y-axis (buccoversion and/or palato/linguoversion) and around the axis of the tooth (Z-axis) were selected most frequently. This is in contrast to rotation around the X-axis (mesial and/or distal angulation), which was selected only once. These findings seem to correlate well with our clinical experience and seem in accordance with the limited available textbook instructions that mostly focus on rotations or movements around the longitudinal axis and buccolingual axis of a tooth (Stegenga 2013). Some of the selected features, on the other hand, are less well understood—for example, the selection of an average torque feature (mesial angulation) that does not match with an unselected rotational feature in the same direction. It might have to do with the position of the teeth; for example, a more mesial angulation is expected in dorsally located teeth, especially if a neighboring (mesial) tooth is absent. The latter has not been taken into account, and these findings need additional analysis in future work.

Due to the pioneering character of this study, no direct comparison is possible with previous literature. The available scientific literature on tooth removal procedures is surprisingly scarce and limited to the evaluation of exerted forces using a variety of methodologies and heterogeneous outcomes (Ahel et al. 2006; Chiba et al. 1980; Dietrich et al. 2020; Lehtinen and Ojala 1980; Sugahara et al. 2021). When this project started, many uncertainties in terms of achievability existed (van Riet et al. 2020). One of the most important downsides to our data set and, therefore, our model is that the data were collected ex vivo. While the participating, experienced, oral- and maxillofacial surgeons considered the fresh-frozen material as clinically representative, it is unknown in what way the freezing process influences the biomechanical properties of tooth removal. This should be taken into account when interpreting our results. Due to the uncertainties that coincide with the development of a novel measurement setup, we aimed for 100 successful experiments on fresh-frozen material. This should be considered a small data set, and its size has a strong effect on the strength of our model. For example, recorded metadata such as periodontal health, root length, or type of surgeon could not be incorporated in this model, nor could differences in outcome be evaluated. Also, radiological metadata were unavailable, which could contain relevant variables, such as bone density, which is preferable to incorporate in future research initiatives.

With currently available technology, it will be very challenging to gain the same quality of data in a clinical situation. Efforts should nevertheless be made to correlate results found in fresh-frozen cadavers to the clinical situation. Moreover, a translation should be made between this theoretical model and clinical use. Two possibilities are discussed. First, improved (evidence-based) preclinical educational methods can be developed. Previous scientific efforts also had educational reasons at heart (Lehtinen and Ojala 1986; Sugahara et al. 2021). The authors are planning to enhance the measurement setup to a much simpler version that is to be used for dental training. Using a force/torque sensor, students are able to receive direct feedback on their performance while practicing on plastic or cadaver models. Results of this study can be used to decide which feedback (or which feature) is most relevant during removal of specific teeth, to optimize force-based learning methods (Hardon et al. 2018). Data from this study might also, in the near future, be useful in the development of virtual learning methods in an evidence-based manner. Second, it could be evaluated if metadata, after enlarging the database, can be used to develop a clinically relevant classification for expected tooth removal complexity. This, potentially, could help the clinician to decide whether referral for an extraction is deemed necessary, based on their own competences.

Concluding, this article discussed the design and performance of a multiclass classification model for tooth removal. Despite the relatively small data set, the quality of the data was sufficient to develop a first model with reasonable performance. The results presented in this article can be considered a strong first step toward an improved understanding of these complex procedures. This improved understanding could potentially aid in the development of evidence-based educational material and clinical guidelines for tooth removal in the near future.

## Author Contributions

W.M. de Graaf, T.C.T. van Riet, contributed to conception and design, data acquisition, analysis, and interpretation, drafted the manuscript; J. de Lange, contributed to conception and design, data acquisition, analysis, and interpretation, critically revised the manuscript; J. Kober, contributed to conception, data acquisition and interpretation, critically revised the manuscript. All authors gave their final approval and agree to be accountable for all aspects of the work.

## Supplemental Material

sj-docx-1-jdr-10.1177_00220345221117745 – Supplemental material for A Multiclass Classification Model for Tooth Removal Procedures Click here for additional data file. Supplemental material, sj-docx-1-jdr-10.1177_00220345221117745 for A Multiclass Classification Model for Tooth Removal Procedures by W.M. de Graaf, T.C.T. van Riet, J. de Lange and J. Kober in Journal of Dental Research

## References

[bibr1-00220345221117745] Ahel V Brekalo I Ahel J Brumini G . 2006. Measurement of tooth extraction forces in upper incisors. Coll Antropol. 30(1):31–35. 16617572

[bibr2-00220345221117745] Bishop CM . 2006. Pattern recognition and machine learning. New York (NY): Springer.

[bibr3-00220345221117745] Brick TR Koffer RE Gerstorf D Ram N . 2017. Feature selection methods for optimal design of studies for developmental inquiry. J Gerontol B Psychol Sci Soc Sci. 73(1):113–123. 28164232 10.1093/geronb/gbx008 PMC6075467

[bibr4-00220345221117745] Bursac Z Gauss CH Williams DK Hosmer DW . 2008. Purposeful selection of variables in logistic regression. Source Code Biol Med. 3:17. 19087314 10.1186/1751-0473-3-17 PMC2633005

[bibr5-00220345221117745] Celsus AC . 1814. Aulus Cornelius Celsus of medicine. In Eight books. Translated with notes critical and explanatory. Book vii. Edinburgh: Dickinson and Company. p. 338.

[bibr6-00220345221117745] Challis JH . 2020. Quaternions as a solution to determining the angular kinematics of human movement. BMC Biomed Eng. 2:5. 32903359 10.1186/s42490-020-00039-z PMC7422562

[bibr7-00220345221117745] Challis RE Kitney RI . 1983. The design of digital filters for biomedical signal processing. Part 3: The design of Butterworth and Chebychev filters. J Biomed Eng. 5(2):91–102. 6855219 10.1016/0141-5425(83)90026-2

[bibr8-00220345221117745] Chiba M Ohshima S Takizawa K . 1980. Measurement of the force required to extract the rat mandibular incisor. Arch Oral Biol. 25(10):683–687. 6940526 10.1016/0003-9969(80)90101-6

[bibr9-00220345221117745] Cicciù M Bramanti E Signorino F Cicciù A Sortino F . 2013. Experimental study on strength evaluation applied for teeth extraction: an in vivo study. Open Dent J. 7:20–26. 23539609 10.2174/1874210601307010020 PMC3606950

[bibr10-00220345221117745] Dietrich T Schmid I Locher M Addison O . 2020. Extraction force and its determinants for minimally invasive vertical tooth extraction. J Mech Behav Biomed Mater. 105:103711. 32279853 10.1016/j.jmbbm.2020.103711

[bibr11-00220345221117745] Hardon SF Horeman T Bonjer HJ Meijerink W . 2018. Force-based learning curve tracking in fundamental laparoscopic skills training. Surg Endosc. 32(8):3609–3621. 29423553 10.1007/s00464-018-6090-7 PMC6061061

[bibr12-00220345221117745] Lehtinen R Ojala T . 1980. Rocking and twisting moments in extraction of teeth in the upper jaw. Int J Oral Surg. 9(5):377–382. 6783563 10.1016/s0300-9785(80)80063-9

[bibr13-00220345221117745] Lehtinen R Ojala T . 1986. Apparatus for simulating extraction forces. Int J Oral Maxillofac Surg. 15(4):444–445. 3091726 10.1016/s0300-9785(86)80035-7

[bibr14-00220345221117745] Luo W Phung D Tran T Gupta S Rana S Karmakar C Shilton A Yearwood J Dimitrova N Ho TB , et al. 2016. Guidelines for developing and reporting machine learning predictive models in biomedical research: a multidisciplinary view. J Med Internet Res. 18(12):e323. 10.2196/jmir.5870 PMC5238707 27986644

[bibr15-00220345221117745] Pedregosa F Varoquaux G Gramfort A Michel V Thirion B Grisel O Blondel M Prettenhofer P Weiss R Dubourg V , et al. 2011. Scikit-learn: machine learning in Python. J Mach Learn Res. 12(85):2825–2830.

[bibr16-00220345221117745] Schwendicke F Singh T Lee JH Gaudin R Chaurasia A Wiegand T Uribe S Krois J ; IADR e-oral health network and the ITU WHO focus group AI for Health. 2021. Artificial intelligence in dental research: checklist for authors, reviewers, readers. J Dent. 107:103610.33631303

[bibr17-00220345221117745] Stegenga B . 2013. Mka chirurgie. Assen: van Gorcum.

[bibr18-00220345221117745] Sugahara K Bessho H Nishiyama A Koyama Y Koyachi M Toyoda T Kasahara K Watanabe A Takano M Katakura A . 2021. The utility of custom-developed tooth extraction simulator—a comparative analysis from beginner to trainer. J Oral Maxillofac Surg Med Pathol. 33(1):43–47.

[bibr19-00220345221117745] van Riet TCT de Graaf WM van Antwerpen R van Frankenhuyzen J de Lange J Kober J . 2020. Robot technology in analyzing tooth removal—a proof of concept. Annu Int Conf IEEE Eng Med Biol Soc. 2020:4721–4727. 33019046 10.1109/EMBC44109.2020.9176405

[bibr20-00220345221117745] van Rossum G Drake FL Jr . 1995. Python reference manual. Amsterdam: Centrum voor Wiskunde en Informatica Amsterdam.

[bibr21-00220345221117745] Yoganathan AP Gupta R Corcoran WH . 1976. Fast Fourier transform in the analysis of biomedical data. Med Biol Eng. 14(2):239–245. 940380 10.1007/BF02478755

[bibr22-00220345221117745] Zhang H . 2004. The optimality of naive bayes. FLAIRS Conference: Proceedings of the Seventeenth International Florida Artificial Intelligence Research Society Conference; Miami Beach, FL.

