# Supplementary material for: A Multiclass Classification Model for Tooth Removal Procedures
Source: J Dent Res. 2022 Sep 9;101(11):1357–62. doi: 10.1177/00220345221117745 (PMC9516607; doi:10.1177/00220345221117745)
Supplement: sj-docx-1-jdr-10.1177_00220345221117745 – Supplemental material for A Multiclass Classification Model for Tooth Removal Procedures [file sj-docx-1-jdr-10.1177_00220345221117745.docx]

***A multiclass classification model for tooth removal procedures***

**W.M. de Graaf, Tom C.T. van Riet, Jan de Lange and Jens Kober**

*Appendix Table 1. An overview of all features designed through a collaboration between a clinician and a computer scientist. AUC = area under the curve, n = number, N = Newton, Ns = Newton second, Nms = Newton meter second, deg = degree, deg/s = degrees per second, (+) = only positive values on specified axis, (-) = only negative values on specified axis, X-axis = buccolingual, Y-axis = mesiodistal, Z-axis = longitudinal axis. X+Y+Z = sum of all axes. In case of rotational data (torques and all rotational data features) a rotation around the mentioned axis takes place.*

| **Force and torque data features** | **Axis** | **Direction** | **n = 42** |
| --- | --- | --- | --- |
| Sum (AUC) of forces (Ns) | X+Y+Z X-axis (+) X-axis (-) Y-axis (+) Y-axis (-) Z-axis (+) Z-axis (-) | all buccal lingual mesial distal intrusion extrusion | 7 |
| Sum (AUC) of torque (Nms) | X+Y+Z X-axis (+) X-axis (-) Y-axis (+) Y-axis (-) Z-axis (+) Z-axis (-) | all mesial angulation distal angulation buccoversion palato-linguoversion mesiopalatal rotation lingual rotation | 7 |
| Average forces (N) | X+Y+Z X-axis (+) X-axis (-) Y-axis (+) Y-axis (-) Z-axis (+) Z-axis (-) | all buccal lingual mesial distal intrusion extrusion | 7 |
| Average torques (Nm) | X+Y+Z X-axis (+) X-axis (-) Y-axis (+) Y-axis (-) Z-axis (+) Z-axis (-) | all mesial angulation distal angulation buccoversion palato-linguoversion mesiopalatal rotation lingual rotation | 7 |
| Peak forces (N) | X+Y+Z X-axis Y-axis Z-axis | all buccal/palatal-lingual mesial/distal intrusion/extrusion | 4 |
| Peak torque (Nm) | X+Y+Z X-axis  Y-axis   Z-axis | all mesial/distal angulation buccoversion/palato-linguoversion mesiopalatal/lingual rotation | 4 |
| Percentage of amount of force, relative to the sum of all three axis (%) | X-axis  Y-axis  Z-axis | buccal/palatal-lingual mesial/distal intrusion/extrusion | 3 |
| Percentage of amount of torque, relative to the sum of all three axis (%) | X-axis  Y-axis   Z-axis | mesial/distal angulation buccoversion/palato-linguoversion mesiopalatal/lingual rotation | 3 |
| **Rotational data features** | **Axis** | **Direction** | **n = 33** |
| Percentage of amount of rotation, relative to the sum of all three axes (%) | X-axis Y-axis Z-axis | mesial-distal angulation bucco-palato/linguoversion mesiobuccal- mesiopalatal/lingual rotation | 3 |
| Variation of rotation on a single axis (deg) | X-axis  Y-axis  Z-axis | mesial-distal angulation bucco-palato/linguoversion mesiobuccal- mesiopalatal/lingual rotation | 3 |
| Maximum rotations (deg) | X-axis (+) X-axis (-) Y-axis (+) Y-axis (-) Z-axis (+) Z-axis (-) | mesial angulation distal angulation buccoversion linguoversion mesiobuccal rotation lingual rotation | 6 |
| Average rotations (deg) | X-axis (+) X-axis (-) Y-axis (+) Y-axis (-) Z-axis (+) Z-axis (-) | mesial angulation distal angulation buccoversion linguoversion mesiobuccal rotation lingual rotation | 6 |
| Average rotational velocity (deg/s) | X-axis (+) X-axis (-) Y-axis (+) Y-axis (-) Z-axis (+) Z-axis (-) | mesial angulation distal angulation buccoversion linguoversion mesiobuccal rotation lingual rotation | 6 |
| Maximum rotational velocity (deg/s) | X-axis (+) X-axis (-) Y-axis (+) Y-axis (-) Z-axis (+) Z-axis (-) | mesial angulation distal angulation buccoversion linguoversion mesiobuccal rotation lingual rotation | 6 |
| Variation of rotational velocity on a single axis (deg/s) | X-axis Y-axis Z-axis | mesial-distal angulation bucco-palato/linguoversion mesiobuccal- mesiopalatal/lingual rotation | 3 |

*Appendix Table 2: base characteristics of experimental material and experiments. mm = millimeter*.

| Base Characteristics | Total number |
| --- | --- |
| Fresh-frozen specimens Upper jaws with teeth Lower jaws with teeth | **7** 6 6 |
| Total number of experiments  Successful experiments: Without complications Boney wall fracture Root fracture (late) Crown fracture/failure (with root removal)  Unsuccessful experiments: Insufficient fixation of jaw Early crown fracture/failure (without root removal) Robot / software errors Excessive slippage of the forceps | **127**  **110** 94 9 6 3  **17** 8 5  3 1 |
| Periodontal state (out of 110 experiments) - sound (pocket depth <3mm) - recessions - mild decay (pocket depth 3-5mm) - severe decay (pocket depth >5mm) | 82 33 16 12 |
| Restorative state (out of 110 experiments) - sound - direct restoration large (≥ 2 surfaces) - indirect restoration - direct restoration small (≤ 2 surfaces) | 47 25 20 18 |
